# Validation of Platelet Counting Accuracy With the Celltac F Automated Hematology Analyzer

**DOI:** 10.1155/JAMMC.2005.235

**Published:** 2005

**Authors:** Yutaka Nagai, Hiroshi Kondo, Noriyuki Tatsumi

**Affiliations:** 1 Nihon Kohden Corporation 1-31-4 Shinjuku Tokyo 161-8560 Japan; 2 Daito Bunka University Saitama 335-8501 Japan; 3 International Buddhist University Osaka 583-8501 Japan

## Abstract

Rapid and accurate analysis of platelet count plays an important role in evaluating hemorrhagic status. Therefore, we evaluated platelet counting performance of a hematology analyzer, Celltac F (MEK-8222, Nihon Kohden Corporation, Tokyo, Japan), that features easy use with low reagent consumption and high throughput while occupying minimal space in the clinical laboratory. All blood samples were anticoagulated with dipotassium ethylenediaminetetraacetic acid (EDTA-2K). The samples were stored at room temperature (18^^^C–22^^^C) and tested within 4 hours of phlebotomy. We evaluated the counting ability of the Celltac F hematology analyzer by comparing it with the platelet counts obtained by the flow cytometry method that ISLH and ICSH recommended, and also the manual visual method by Unopette (Becton Dickinson Vacutainer Systems). The ICSH/ISLH reference method is based on the fact that platelets can be stained with monoclonal antibodies to CD41 and/or CD61. The dilution ratio was optimized after the precision, coincidence events, and debris counts were confirmed by the reference method. Good correlation of platelet count between the Celltac F and the ICSH/ISLH reference method (*r* = 0.99, and the manual visual method (*r*= 0.93) were obtained. The regressions were *y* = 0.90 x+9.0 and *y*=1.11x+8.4, respectively. We conclude that the Celltac F hematology analyzer for platelet counting was well suited to the ICSH/ISLH reference method for rapidness and reliability.


Journal of Automated Methods & Management in Chemistry, **2005** (2005), no. 4, 235–239
© 2005 Hindawi Publishing Corporation

### Validation of Platelet Counting Accuracy With the Celltac F Automated Hematology Analyzer

Yutaka Nagai,[1] Hiroshi Kondo,[2] and Noriyuki Tatsumi[3]

[1] *Nihon Kohden Corporation 1-31-4, Shinjuku, Tokyo 161-8560, Japan*
[2] *Daito Bunka University, Saitama 335-8501, Japan*
[3] *International Buddhist University, Osaka 583-8501, Japan*

*Received 25 May 2005; Accepted 6 June 2005*

Rapid and accurate analysis of platelet count plays an important role in evaluating hemorrhagic status. Therefore, we evaluated platelet counting performance of a hematology analyzer, Celltac F (MEK-8222, Nihon Kohden Corporation, Tokyo, Japan), that features easy use with low reagent consumption and high throughput while occupying minimal space in the clinical laboratory. All blood samples were anticoagulated with dipotassium ethylenediaminetetraacetic acid (EDTA-2K). The samples were stored at room temperature (18°C–22°C) and tested within 4 hours of phlebotomy. We evaluated the counting ability of the Celltac F hematology analyzer by comparing it with the platelet counts obtained by the flow cytometry method that ISLH and ICSH recommended, and also the manual visual method by Unopette (Becton Dickinson Vacutainer Systems). The ICSH/ISLH reference method is based on the fact that platelets can be stained with monoclonal antibodies to CD41 and/or CD61. The dilution ratio was optimized after the precision, coincidence events, and debris counts were confirmed by the reference method. Good correlation of platelet count between the Celltac F and the ICSH/ISLH reference method ($r = 0.99$), and the manual visual method ($r = 0.93$) were obtained. The regressions were $y = 0.90x + 9.0$ and $y = 1.11x + 8.4$, respectively. We conclude that the Celltac F hematology analyzer for platelet counting was well suited to the ICSH/ISLH reference method for rapidness and reliability.

### 1. INTRODUCTION

Modern highly automated hematology analyzers have adequate reproducibility, but there are concerns over accuracy. For example, it is well known that erythrocyte fragments or microcytes cause a pseudo increase in platelet counts [1, 2].

The recently developed Celltac F (MEK-8222, Nihon Kohden, Tokyo, Japan) is a new hematology analyzer that has an excellent reproducibility, a wide linearity range of platelet counting, and a good correlation with CD-4000 (Abbott, Abbott Park, Ill, USA) [3, 4].

In this study, we evaluated the ability of the Celltac F hematology analyzer to count platelets accurately in comparison to the newly published flow cytometric reference method from the International Council for Standardization in Hematology (ICSH)/International Society for Laboratory Hematology (ISLH) [5]. This method is based on the fact that platelets can be stained with monoclonal antibodies to CD41(GPIIb) and/or CD61(GPIIIa). We also evaluated precision, stability, and final total dilution in the proposed ICSH/ISLH reference method.

Correspondence and reprint requests to Yutaka Nagai, Nihon Kohden Corporation 1-31-4, Shinjuku, Tokyo 161-8560, Japan E-mail: yutaka_nagai@mb6.nkc.co.jp.

### 2. MATERIALS AND METHODS

### 2.1. Blood samples

All blood samples were anticoagulated with dipotassium ethylenediaminetetraacetic acid (EDTA-2K) and were obtained after informed consent from healthy adult volunteers or as the residual material from patient specimens collected for clinical testing. All blood samples were stored at room temperature (18–22°C) and tested within 4 hours of phlebotomy.

### 2.2. Automated platelet counts

*Instrument*

The Celltac F has an impedance counter for platelet and red blood cells (RBCs). It use pulse-editing circuitry in combination with a chamber which reduces the recirculation of cells, and the total electronic dead time for a signal pulse exceeding the lower threshold is minimized by high-speed digital signal processing. The inherent problems of coincident passage and influence of recirculation of cells during the measurement process [6] are minimized by the new technology, which automatically detects the position between platelet distribution and RBC distribution from the histogram. Normal blood shows a clear separation in the platelet volume range and RBC volume range (Figure 1a). When abnormal blood with

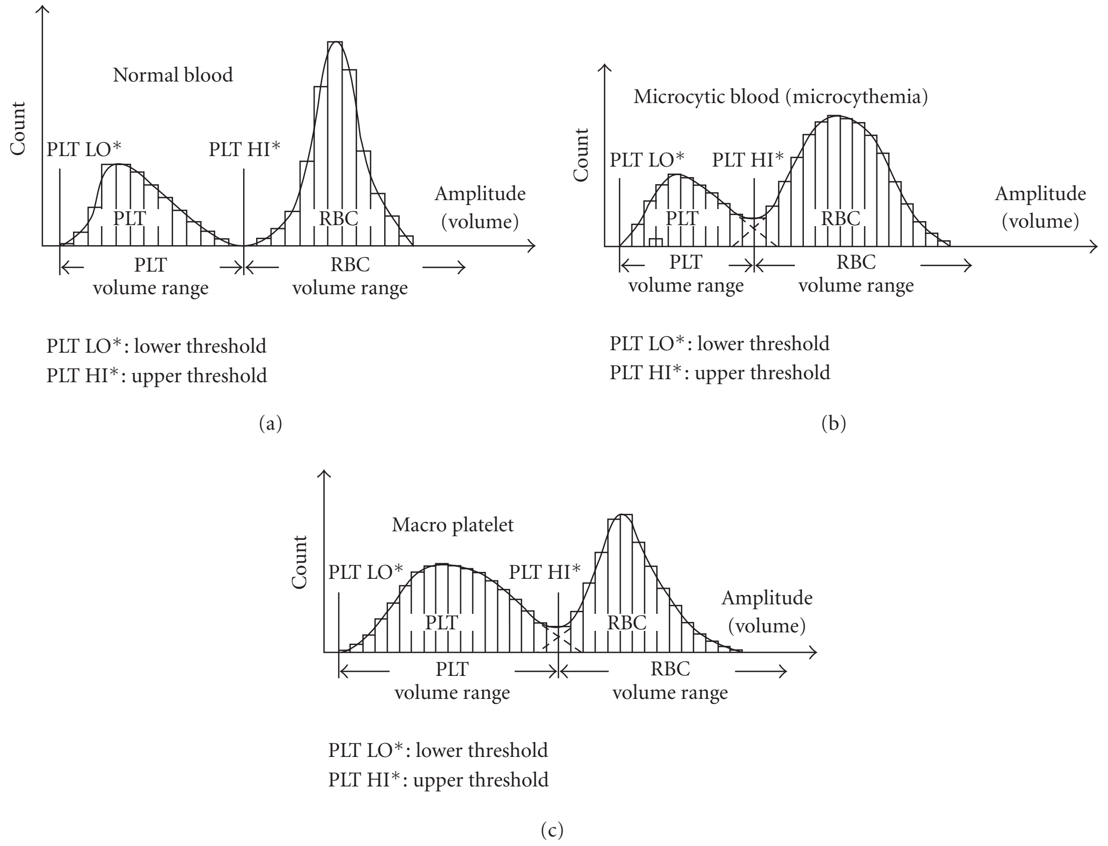

Figure 1: Platelet/red blood cell histogram (a) normal sample, (b) micro RBC, (c) large platelet.

micro RBC or large platelets are counted, the upper threshold position of platelet distribution is set to the optimized position for accurate counting (Figures 1b and 1c). The influence of coincidence between the next pulse and the previous pulse can be minimized by high-speed digital signal processing. Therefore, the accuracy is maintained at a high level.

*Reagents*

Manufacturer-recommended reagents were used.

*Calibrations and controls*

The instruments were calibrated according to the manufacturer's guidelines [7]. Three levels of control material (low, normal, and high) were used.

### 2.3. ICSH/ISLH procedure [5]

The specimens were mixed 8 times by gentle complete inversion. 5 $\mu$L of blood in 100 $\mu$L phosphate buffered saline (PBS) containing 0.1% bovine serum albumin (BSA) (Sigma Diagnostics, Mississauga, Canada) and 10 $\mu$L of 1:1 CD41-fluorescein isothiocyanate(FITC)/CD61-FITC (Beckman Coulter, Miami, Fla) cocktail were combined in a polypropylene tube. The mixed solution was allowed to incubate for 15 minutes in the dark at room temperature. After incubation, 900 $\mu$L PBS containing 0.1% BSA was added to the tube. The diluted mixture was transferred to a tube containing PBS to make a final dilution. The sample was covered with parafilm and mixed by gentle inversion. The samples were then analyzed on a Coulter EPICS-XL flow cytometer with bitmap analysis regions [9]. The flow rate was set to medium. The stop count was set to 50 000 red blood cell (RBC) events, or 1000 or 2000 platelet events, and the upper bound of the measurement time was set to 300 seconds. Platelet : RBC ratios were determined for each sample, and the ratio was corrected for platelet-RBC coincidence. The platelet count was derived by multiplying the ratio by the RBC count obtained from the Celltac F hematology analyzer which is based on the reference method of ICSH [8].

### 2.4. Study design

*Study A*

We assessed the measurement condition of the ICSH/ISLH reference method.

*Precision.* Five fresh blood samples were used to estimate the "within-run precision" for the clinical range of interest. We also assessed the effects of RBC counts on the CV for platelet counts. Platelet-rich plasma, platelet-poor plasma, and packed RBC of the same person were prepared by centrifugation. Three kinds of RBC concentration samples were prepared by packed RBC dilution with platelet-poor plasma. After platelet-rich plasma and the RBC samples were

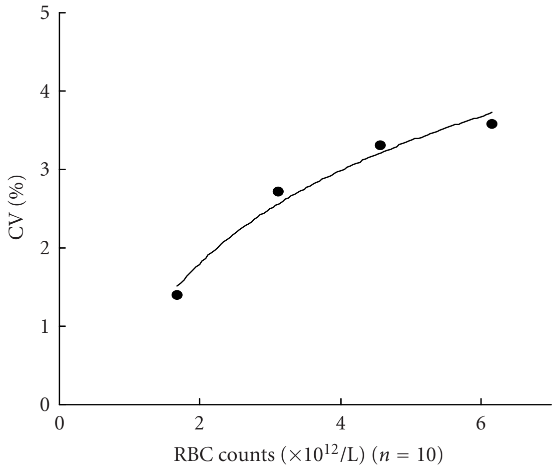

Figure 2: The CV of platelet counts in the sample containing four levels of red blood cell counts using the ICSH/ISLH reference method.

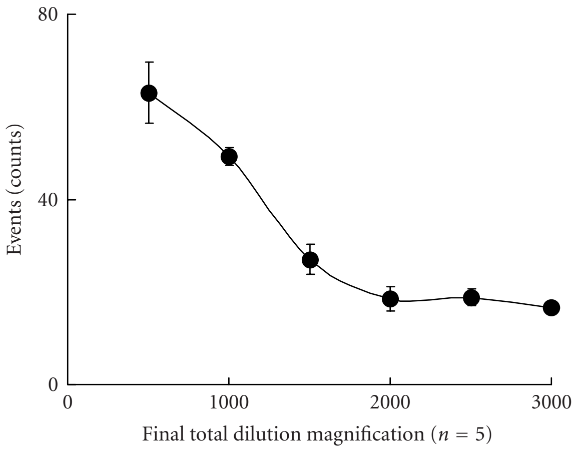

Figure 3: Coincidence events for the ICSH/ISLH reference method when final total dilution of the blood sample increased.

mixed at the same rate, the number of platelets in the mixture was measured. These samples were run 6 times. The SD and coefficients of variation (CV) were calculated. In the CV examination of Study A, the stop count was set to 50 000 red blood cell (RBC) events, 2000 platelet events, and the upper bound of the measurement time was set to 300 seconds.

*Sample Stability.* Blood was collected from six healthy donors and stored at room temperature. The samples were analyzed at 0, 2, 4, and 6 hours after collection.

### *Study B: platelet count correlation*

Platelet count performance was evaluated in comparison with the ICSH/ISLH reference method and the manual visual method by Unopette (Becton Dickinson Vacutainer Systems, Franklin Lakes, NJ).

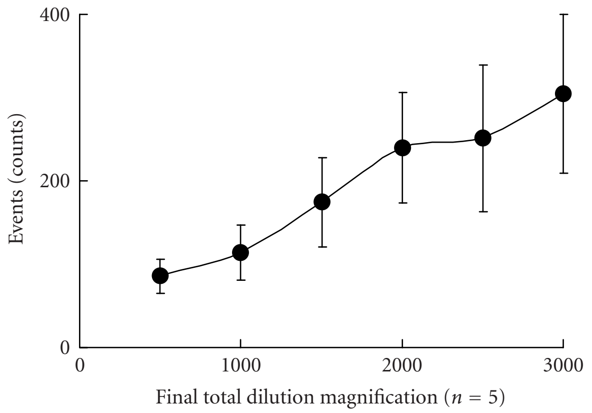

Figure 4: Debris events for the ICSH/ISLH reference method when final total dilution of the blood sample increased.

### *2.5. Statistical analysis*

Two-way analysis of variance, signed rank test, correlation and regression analysis using Stat View Japanese edition (SAS institute Inc) were performed on the results. Probability values of less than .05 (two-tailed) were regarded as significant. All values are presented as mean ± SD.

### 3. RESULTS

### *3.1. Study A*

The CV from within-run replicate analysis of a normal blood sample using the ICSH/ISLH reference method was 3.2% ($n = 10$). To confirm the effect of RBC counts on the CV of platelet counts, three kinds of samples with different RBC counts were analyzed 6 times by the ICSH/ISLH reference method. Though CV of platelet counts was 4% or less, it tended to increase with an increase in RBC count. (Figure 2).

Stability studies showed that platelet count by the ICSH/ISLH reference method was stable with no significant difference for 6 hours at room temperature.

Figures 3 and 4 show the changes of coincidence counts and debris counts by the ICSH/ISLH reference method when final total dilution of the blood sample increased. The coincidence events decreased and the debris counts increased with increasing final total dilution. We selected 1500 times as the final total dilution for the following experiments.

### *3.2. Study B*

The correlation of the Celltac F platelet count to the ICSH/ISLH reference method and the manual visual method are presented in Figures 5 and 6. The correlation coefficients ($r$) for the Celltac F versus the ISLH/ICSH reference method and the manual visual method were 0.99 and 0.93 ($n = 45$), respectively.

### 4. DISCUSSION

Hematology laboratories continue to face high demands for accurate platelet analysis, especially in the low range used for

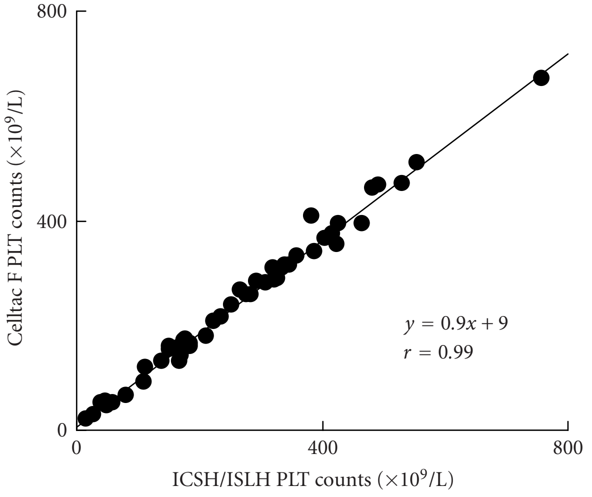

Figure 5: The correlation of the Celltac F platelet counts to the ICSH/ISLH reference method.

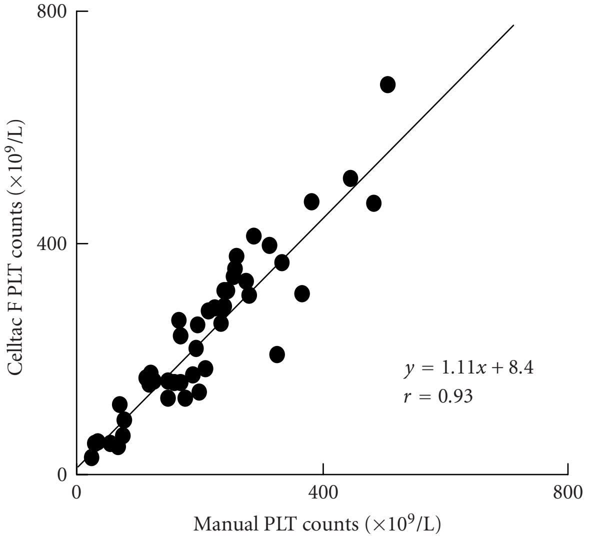

Figure 6: The correlation of the Celltac F platelet counts to the manual visual method.

making therapeutic decision. The ICSH/ISLH platelet enumeration standard was developed as a reference method for comparison of platelet counting technology [5], suitable for use in the reference laboratory. We evaluated the precision, stability, and final total dilution of this method.

Platelet counts by the ICSH/ISLH reference method showed acceptable stability for 6 hours, but the result of precision tests showed that CV was influenced by the erythrocyte counts. All measurements were stopped by the upper bound of the platelet events. The CV increased with increasing RBC events, which depended on the dilution rate. The coincident events of RBC and debris events increased according to an increase in RBC events, and the number of debris events was more than that of coincident events. The debris events increased while coincident events decreased when the dilution rate of the sample increased. The ICSH/ISLH reference method may count bubbles [10] and dust, and so forth, as debris events. To minimize the influence of debris events or coincident events, the dilution rate was set to 1500 times. The measuring time of a normal sample was approximately 20 seconds, and the number of measurement events was changed from 1000 counts to 1500 counts per second. This measurement condition was appropriate, because the number of events tends to decrease after approximately 30 seconds.

This method is not considered a routine test in the clinical laboratory due to cost- and time-consuming sample-making process. Automated hematology analyzers are used in a routine test in the clinical laboratory; therefore, a comparison with the reference method is very important. It has already been reported that the platelet counts obtained from the Celltac F have a good correlation with both impedance count and optical count obtained from the CD-4000, especially in a low range of platelets [3]. Although our former study showed that the Celltac F allows high-speed processing in a compact machine, we were not able to evaluate the accuracy of platelet counting by Celltac F using the ICSH/ISLH reference method [5]. The comparison of results between the Celltac F and the ICSH/ISLH reference method showed excellent correlation to platelet counts. The comparison of results between the Celltac F and the manual visual method also showed excellent correlation to platelet counts if we consider the reproducibility of the manual visual method. The Celltac F is a very efficient hematology analyzer; therefore, it can satisfy routine laboratory tests.

### 5. CONCLUSION

In conclusion, Celltac F is a very efficient hematology analyzer providing accurate platelet counts and is a highly functional small-sized analyzer. It has a high potential to contribute to cost-effective laboratory management.

### REFERENCES

[1] J. Hammerstrom, "Spurious platelet counts in acute leukaemia with DIC due to cell fragmentation," *Clinical and Laboratory Haematology*, vol. 14, no. 3, pp. 239–243, 1992.

[2] A. M. Akwari, D. W. Ross, and S. A. Stass, "Spuriously elevated platelet counts due to microspherocytosis," *American Journal of Clinical Pathology*, vol. 77, no. 2, pp. 220–221, 1982.

[3] H. Kondo, N. Tatsumi, and Y. Nagai, "Accurate measurement of low platelet count and low white blood cell count using a compact-type hematology analyzer, Celltack F," in *Proc. 22nd World Congress of Pathology & Laboratory Medicine (WASPaLM '03)*, pp. 231–235, Busan, South Korea, August–September 2003.

[4] H. Kondo, T. Akiyama, N. Tatsumi, and Y. Nagai, "Performance evaluation of the complete blood count and white blood cell differential parameters obtained using a Celltack F automated hematology analyzer," *Laboratory Hematology*, vol. 10, no. 1, pp. 3–13, 2004.

[5] International Council for Standardization in Haematology Expert Panel on Cytometry International Society of Laboratory Hematology Task Force on Platelet Counting, "Platelet counting by RBC/platelet ratio method," *American Journal of*

*Clinical Pathology*, vol. 115, no. 3, pp. 460–464, 2001, a reference method.

[6] W. Groner and E. Simson, "Technology fundamentals," in *Practical Guide to Modern Hematology Analyzers*, W. Groner and E. Simson, Eds., p. 21, John Wiley & Sons, New York, NY, USA, 1995.

[7] Nihon Kohden Celltack F (MEK-8222) Demonstration Guide, Nihon Kohden Corporation, Tokyo, Japan, 2003.

[8] International Committee for Standardization in Haematology; Expert Panel on Cytometry, "The assignment of values to fresh blood used for calibrating automated blood cell counters," *Clinical and Laboratory Haematology*, vol. 10, no. 2, pp. 203–212, 1988.

[9] M. Keeney, W. Brown, and I. C. Yee, "Platelet counts and flagging rates from the LH 750 hematology analyzer compared with the ICSH/ISLH platelet reference method and the gen-S hematology analyzer," *Laboratory Hematology*, vol. 7, no. 4, pp. 204–210, 2001.

[10] C. Tanaka and K. Fujimoto, "The reference method for platelets enumeration," *Sysmex Journal Web*, vol. 1, suppl. 2, pp. 35–39, 2000.

